# Effect of Play-based Therapy on Meta-cognitive and Behavioral Aspects of Executive Function: A Randomized, Controlled, Clinical Trial on the Students With Learning Disabilities

**DOI:** 10.18869/nirp.bcn.8.3.203

**Published:** 2017

**Authors:** Samaneh Karamali Esmaili, Narges Shafaroodi, Afsoon Hassani Mehraban, Akram Parand, Masoume Zarei, Saeed Akbari-Zardkhaneh

**Affiliations:** 1. Department of Occupational Therapy, School of Rehabilitation Sciences, Iran University of Medical Sciences, Tehran, Iran.; 2. Department of Psychology, Faculty of Psychology and Education, University of Tehran, Tehran, Iran.; 3. Department of Psychology, Faculty of Education and Psychology, Shahid Beheshti University, Tehran, Iran.

**Keywords:** Executive function, Learning disability, Play

## Abstract

**Introduction:**

Although the effect of educational methods on executive function (EF) is well known, training this function by a playful method is debatable. The current study aimed at investigating if a play-based intervention is effective on metacognitive and behavioral skills of EF in students with specific learning disabilities.

**Methods:**

In the current randomized, clinical trial, 49 subjects within the age range of 7 to 11 years with specific learning disabilities were randomly assigned into the intervention (25 subjects; mean age 8.5±1.33 years) and control (24 subjects; mean age 8.7±1.03 years) groups. Subjects in the intervention group received EF group training based on playing activities; subjects in the control group received no intervention. The behavior rating inventory of executive function (BRIEF) was administered to evaluate the behavioral and cognitive aspects of EF. The duration of the intervention was 6 hours per week for 9 weeks. Multivariate analysis of covariance was used to compare mean changes (before and after) in the BRIEF scores between the groups.

**Results:**

The assumptions of multivariate analysis of covariance were examined. After controlling pre-test conditions, the intervention and control groups scored significantly differently on both the metacognition (P=0.002; effect size=0.20) and behavior regulation indices (P=0.01; effect size=0.12) of BRIEF.

**Conclusion:**

Play-based therapy is effective on the metacognitive and behavioral aspects of EF in students with specific learning disabilities. Professionals can use play-based therapy rather than educational approaches in clinical practice to enhance EF skills.

## 1. Introduction

Specific Learning Disability (SLD) is a minimal brain damage. Children with SLD have deficits in reading, writing, and mathematics, while the intelligent quotient is normal and training opportunities are adequate (American Psychiatric Association, 2013). The core problem in SLD is poor academic achievement. Many studies showed deficits in EF in students with SLD (Hooper, Swartz, Wakely, de Kruif, & Montgomery, 2002; Moura, Simões, & Pereira, 2014; Toll, Van der Ven, Kroesbergen, & Van Luit, 2011; Varvara, Varuzza, Sorrentino, Vicari, & Menghini, 2014) and several proved the link between academic skills and EF (Toll et al., 2011; Varvara et al., 2014). EF as a high-level cortical function is impaired in the students with SLD (Lazar & Frank, 1998).

EF, due to the application of a supervisory role on lower level cognitive processes, causes adaptive human behavior in certain situations. These situations occur in the conditions in which automatic behavior may be insufficient. Some components of EF include inhibition, flexibility, working memory, planning, and monitoring (Ward, 2006). Deficits of EF in children may be observed as deficits in areas such as communication/social interaction, completing assignments at school, doing a craft task or a project, and playing in groups (Fischer & Daley, 2007; Meltzer & Krishnan, 2007). These areas are everyday activities of the child called occupation. Occupation is a dynamic experience comprising of self-organized and self-determined actions directed to the pursuit of the fulfillment of his/her life (such as play/leisure, self-care, and work) (Lazzarini, 2004). EF is the most critical cognitive function that affects participation in everyday occupations (Cramm, Krupa, Missiuna, Lysaght, & Parker, 2013). Cramm et al., (2013) indicated that the assessment and intervention of EF is usually focused on the components such as working memory and attention versus executing occupational performance.

Few clinical trials were found for the dysexecutive function in students with SLD. Mir Mehdi, Alizadeh and Seif-Naraghi (2009) studied the effect of EF training in 4 components of planning, organizing, working memory, and inhibition on improving, reading, and mathematics performance of students with SLD. The intervention program in their study was in the form of instructional tasks using training cards. In another clinical trial, Horowitz-Kraus (2013) strengthened some EF skills including auditory, visual, and cross modality working memory skills. He conducted an intervention with a computerized cognitive program using CogniFit Personal Coach (CPC). Both of the mentioned trials were in the form of structured training, without applying motivation and excitement, both of which are important factors in EF (Pessoa, 2009).

The study by Staiano, Abraham, and Calvert (2012) was the only survey in which the intervention method was not defined in a structured task and included the parameters of excitement and motivation. They investigated the effect of competitive and cooperative exergame play on EF in adolescents without disorder. They showed that the group who experienced a play intervention developed better abilities with respect to EF than the no-play control group. The intervention in their study was the Wii EA Sports Active™ exergame, a computer game with gross movements. Outcomes were measured in these clinical trials by administrating some neuropsychological batteries and computerized tools such as the Delis–Kaplan Executive Function System (D–KEFS), the computerized version of Wisconsin Card Sorting Test (WCST), and the Cornoldy working memory test.

The current literature review showed that in the assessment and treatment of EF in SLD, the focus was typically on the components of performance. In the mentioned clinical trials (except the last one, which was not conducted in students with SLD), the treatment was provided as training EF components. In general, all methods of increasing EF are defined in educational styles and the components of EF are trained apart from each other in these methods (Diamond & Lee, 2011). Keeping training components separate showed the bottom-up approach to EF (Wilding & Whiteford, 2007). Depending on the type of intervention or assessment, a stimulus-driven bottom-up process or a behavior-driven top-down process is enhanced. It affects the quality of the responses. Neuronal responses are often dynamically influenced by a top-down process, and more plasticity happens in a top-down process (Li & Gilbert, 2009). Furthermore, the type of education commonly used as a method to train EF in students with SLD is not fun, and thus, students’ interest, excitement, and motivation to engage with it is low. Motivation and excitement are important factors to determine the type of processing; these 2 elements can affect the perceptual and executive competition at the micro level. It means that positive motivation or emotions increase sensory representation in the brain and improve cognitive and EFs of neural populations (Pessoa, 2009).

Among the activities that people do in their lives, play, with its feature of intrinsic motivation, is an efficient tool that applies EF skills (Shaheen, 2014). It is proven that more areas of the brain are activated when children are engaged in meaningful whole task versus parts of the tasks (Hilton, 2015). It seems that play, as a meaningful occupation, can help in the development of EF skills. A play-based intervention is not studied to strengthen EF in students with SLD. In the current study, it was decided to choose an assessment tool and a treatment method associated with occupational areas to study EF. According to the importance of the peer play in middle childhood, and the limited experience of students with SLD in group play (Wiener, 2004), the group therapy was selected. Therefore, in the current study, an intervention was designed as play sessions with the peers. The impact of this intervention on cognitive and behavioral aspects of EF was measured. It was hypothesized that the peer play can increase the metacognitive and behavioral regulation aspects of EF in students with SLD.

## 2. Methods

### 2.1. Trial design

The current study was a single (assessor)- blinded, randomized, clinical trial. The subjects were randomly assigned into the intervention (25 subjects) and control (24 subjects) groups. Ethical approval was obtained from the Ethical Committee of Iran University of Medical Sciences (IUMS). The trial was registered in the Iranian Registry of Clinical Trials (IRCT). After an informative interview and investigating the inclusion criteria, the subjects who agreed to participate were included in the study. The parents of the participants signed the written informed consent.

### 2. 2. Participants and settings

**Figure 1.**
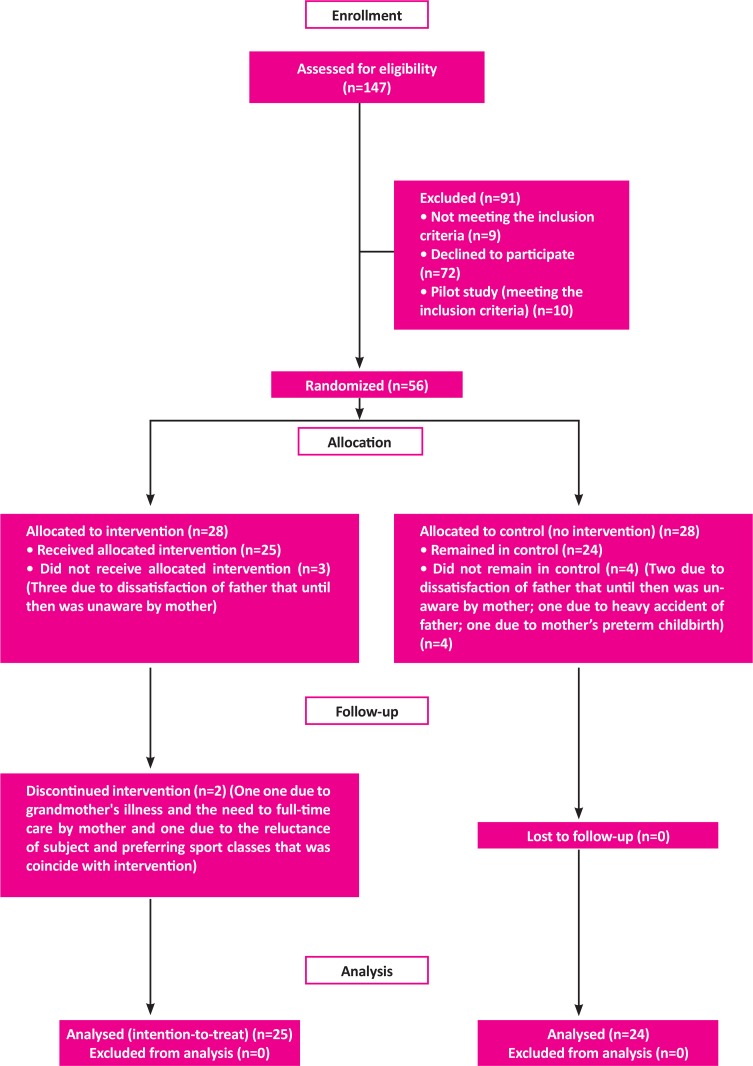
Participant flow.

A total of 49 students with SLD, aged 7 to 11 years were selected. Figure 1 shows the disposition of the entire sample. They were recruited from Educational and Rehabilitation Centers for Specific Learning Difficulties in Tehran, Iran. The subjects were referred to these centers from public schools. The inclusion criteria were as follows: 1) The diagnosis of SLD by a psychiatrist; 2) The literacy of parents to read the questionnaires; and 3) Lack of comorbid psychiatric disorder, as measured by the Persian version of Child Symptom Inventory-4 (CSI-4) and referring to the psychiatrist if there were significant symptoms in CSI-4.

### 2. 3. Intervention

Intervention was performed in groups of 3 to 5 students for 9 weeks, every day from Monday to Thursday in July, August, and September, 2015. The sessions took place at the Rehabilitation School of Iran University of Medical Sciences. The mean number of sessions was 17.2 and the mean length of each session was 165 minutes. Every day, two groups of 3–5 students were treated. The intervention protocol contained the play activities. These activities were selected by reviewing the play history of participants and some books about the children’s play. The selected play activities were analyzed in terms of EF components.

The protocol was supervised and validated by 5 professors of occupational therapy, psychology, and neuroscience who were experts in the field of SLD. In each session, numerous play activities were conducted based on the goals of the session. Session goals contained EF components including inhibition, shifting, emotional control, working memory, initiation, planning, organization of materials, and monitoring. In early sessions, aimed at using the children’s interest, motivation, and activities, which were well-liked and familiar to the children based on their play history were conducted and adapted according to the goals of the session. New play activities were gradually added, and the complexity of performing them graded and increased based on an analysis of the components. Thus, over time, more EF components were gradually involved. The goals of the early sessions contained one of the EF skills, and the goals of the last sessions contained several skills together. Two therapists (an occupational therapist and a psychologist) were responsible to implement the program.

They had been trained to provide the program of each session by the trial manager. To ensure that the program delivery was optimal, the trial therapists were observed, while delivering the program and given feedback on their performance. The sessions were recorded using Closed-Circuit Television (CCTV), and this footage was reviewed by the trial manager. The personal requirements of the children were considered in the implementation of the activities, with steps taken such as assisting certain children with a part of an activity, providing relaxation training, providing anger management training (particularly in sessions whose goal was emotional control), and accommodating a child’s request to visit his/her mother. The performance of participants in each play activity was recorded in the performance recording sheet and was discussed at the daily meetings of the trial manager and the therapists.

### 2. 4. Instruments

The CSI-4 (parent form) was used to screen behavioral and emotional symptoms and determine comorbid disorders. It was a 4-point Likert scale that demonstrated how often the psychiatric symptoms were observed in the students (Sprafkin, Gadow, Salisbury, Schneider, & Loney, 2002). Test-retest reliability of the Persian version of CSI-4 for 11 disorders was 0.29 to 0.76. Except for social phobia, all disorders were significant at the level of 0.01 (Mohammad Esmail & Alipoor, 2002).

The play history interview was a semi-structured interview conducted with the child’s parents. It evaluated the interests, experiences, and play opportunities in 4 areas of play including materials, actions, playmates, and the time/place. This instrument is usually used as an intervention guideline in research and clinical practice (Behnke & Fetkovich, 1984; Bryze, Parham, & Fazio, 2008; Takata, 1969). The qualitative data from the play history interview were used to understand the participants’ play abilities, interests, and motivation to plan the sessions of the intervention.

The BRIEF was used as the outcome measurement in the current study. This tool presents questions about the child’s behavior in the context of natural life and makes the metacognitive and behavioral assessment of EF at home and school possible. The questionnaire is designed for a wide age range of children, from 5 to 18 years, and for a number of disorders such as learning disabilities. It contains 86 items divided into 8 separate subscales. The total score of these subscales is named the Global Executive Composite (GEC). The 8 subscales are divided into 2 broad scales for scoring. The 3 subscales including inhibition, shifting, and emotional control make up the behavior regulation index (BRI), and the remaining 5 subscales including working memory, initiation, plan/organize, organization of materials, and monitoring make up the metacognition index (MCI) (Gioia, Isquith, Guy, & Kenworthy, 2000).

The internal consistency of BRIEF showed the Cronbach’s alpha 0.80 to 0.90 for both parent and teacher forms. The test-retest coefficients in the BRI, MCI, and GEC scales were 0.81, 0.83, and 0.80, respectively in the parent form, and 0.91, 0.90, and 0.92, respectively in the teacher form (Gioia et al., 2000). In examining the internal consistency of the Persian version, Cronbach’s alpha coefficient for GEC was 0.86. The Pearson correlation coefficients for BRI, MCI, and GEC were 0.83, 0.84, and 0.88, respectively (Mohammad Thaqi, Alizadehzarei, Hasani-Mehraban, & Akbarfahimi, 2015).

### 2. 5. Statistical analysis

The statistical analysis was based on the intention-to-treat analysis. The normal distribution of the data was investigated with the Shapiro–Wilk test. The continuous and categorical data were calculated as mean±standard deviation (SD) and frequency, respectively. A change of scores from the beginning to the end of the study was calculated in both groups and were compared using multivariate analysis of covariance (MANCOVA) between the groups. The effect sizes were calculated based on the partial eta squared estimation that is the most frequently reported when using MANCOVA (Cohen, 1973; Levine, 2002). The statistical analysis was conducted using SPSS version 19, and the level of significance was determined <0.05.

## 3. Results

Table 1 shows the baseline characteristics of the subjects by groups. The baseline characteristics showed no significant differences between the groups (P>0.05). The mean changes of the 2 groups before and after the intervention are presented in Table 2. It can be observed that the means of the BRI and MCI indices of BRIEF significantly reduced in the intervention group. The lower score in BRIEF represents less impairment in EF. There was little change in the mean scores between the pre- and post-test measurements in the control group (BRI=0.16; MCI=1.04), but a large change in the intervention group (BRI=8.52; MCI=15.32).

**Table 1.** Baseline characteristics of the study groups.

		**Intervention Group (N=25)**	**Control Group (N=24)**
Age (years)		8.5(1.33)	8.77(1.03)

Gender^ii^	Male	18(72)	17(70.8)
	Female	7(28)	7(29.2)
Grade^ii^	First	8(32)	3(12.5)
	Second	5(20)	8(33.3)
	Third	6(24)	5(20.8)
	Forth	3(12)	7(29.2)
	Fifth	3(12)	1(4.2)
Siblings^ii^	No sibling	8(32)	5(20.8)
	One	14(56)	14(58.3)
	Two	3(12)	5(20.8)

i. Data are expressed as mean (SD)

ii. Data are expressed as number (percent)

**Table 2.** Descriptive statistics of the intervention and control groups in pretest and posttest.

**Scales**	**Group**	**Pretest (Mean±SD)**	**Posttest (Mean±SD)**	**Mean Difference**
Behavior regulation index	Intervention (n=25)	55.88±11.08	47.36±10.08	8.52
	Control (n=24)	54.58±11.05	54.42±10.84	0.16
Metacognition index	Intervention (n=25)	98.60±15.85	83.28±19.65	15.32
	Control (n=24)	96.71±13.62	95.67±12.97	1.04

Before performing the data analysis to investigate the research hypothesis, the assumptions of MANCOVA were examined. All data were normally distributed with the Shapiro–Wilk test. The charts of data bivariate distribution showed a linear relationship between the dependent variables. The tolerance coefficient of BRI and MCI ranged between 0.35 and 0.39. The Variance Increasing Factor (VIF) ranged between 2.59 and 2.86. All these indices were in the acceptable range. The results of the Box tests of equality of covariance matrices (P<0.05; df=3, 421556.14; F=3.03; Box=9.54) and the Leven test of equality of error variance (0.06<F<0.57) suggested the possibility of homogeneity of variances assumptions and multivariate analysis of covariance.

Based on the results shown in Table 3, the F values between group variables of BRI and MCI were 11.16 and 6.24, respectively. Data analysis showed that after controlling the pre-test differences, the profiles of BRIEF of the groups were significant (THotelling=0.25; F=5.51; df=2, 44; P<0.05; η^2^=0.20). The intervention effect size was 0.20 for MCI and 0.12 for BRI. These indices were also based on the Cohen criteria (Cohen, 1973) in the middle to large category.

**Table 3.** Results of the multivariate analysis of covariance between the study groups.

**Scales**	**Sum of Squares**	**Degree of Freedom**	**F**	**P**	**Effect Size**
Behavior regulation index	2187.19	1	11.16	0.01	0.12
Metacognition index	547.96	1	6.24	0.002	0.20

After controlling the pretest differences, the intervention and control groups were significantly different in terms of both the BRI (P<0.05) and MCI (P<0.05) indices of BRIEF. This finding means that intervention could lead to a significant decrease in both scales. The effect size index showed that the intervention had the greatest influence on BRI scale.

## 4. Discussion

The current study aimed at increasing EF in students with SLD by a play-based intervention conducted as group sessions. The obtained results suggested that students with SLD achieved significant changes in EF compared with the control group who did not experience any interventions. There were changes both in the cognitive and behavioral scales of BRIEF. The effect size of play-based therapy in the present study was higher than those of other interventions on students with SLD.

In a meta-analysis on clinical trial studies on SLD, Swanson and Hoskyn (1998) reported that the effect size of interventions on outcomes such as metacognition, problem solving, and social skills that are similar to the current study outcomes were average and relatively small; these studies were educational, and not the play-based interventions. The current study was among the first to report the use of play-based intervention for EF; thus, comparison of the current study with other studies was limited, and explanation of the findings was discussed in detail.

The play-based therapy for EF resulted in significant changes in the intervention group compared to the control group. It suggested that the planning of treatment goals in a playful way can be successful as a treatment for EF. The findings of the present study were consistent with those of Staiano, Abraham, and Calvert (2012), who conducted the only previous study with play intervention on EF, in which they investigated the impact of computerized motion play (e.g. Xbox Kinect™ games) on EF skills in children without disabilities. The intervention of the current study was similar to that of Staiano et al. (2012), but the method of assessing the outcomes was different. The outcome of the study by Staiano et al. (2012) was evaluated using a neuropsychological tool, which had a learning effect on the participants. In addition, their evaluation was conducted immediately after the last session, which made it likely that the findings were simply the result of the final treatment sessions.

One of the strengths of the current study was that the outcome was measured 2 weeks after the intervention (the subjects did not participate in any therapy or class during this time) to ensure that the findings were not just the result of the final session, but were cumulatively derived from the whole program. In the study by Staiano et al. (2012), high-level cognitive skills such as monitoring, organization of materials, and working memory were not evaluated. In their study, planning was interpreted as a sequencing at the level of motor components, and not as stages related to an activity. The current study measured all these components.

Among various interventions, playful interventions applying motivational factors in learning were more effective to enhance EF (Shaheen, 2014). Several studies reported on the interactions between emotion and specific cognitive processes (Pessoa, 2009; Phelps, 2006). Emotion and motivation affect both perceptual and executive neural competition (Pessoa, 2009). The interventions in the current study, focusing on play, were designed to ensure that children were motivated to participate in the treatment (Rodger & Ziviani, 1999); this made the desired outcome of an increase in EF skills far more likely than in the studies that did not use play-based therapies.

The use of BRIEF as an instrument to measure EF in the present study had some advantages, compared with the measurements conducted in similar studies. Typically, the outcome measurements in clinical trial studies on EF were the computerized and neuropsychological tools (Horowitz-Kraus, 2013; Mir Mehdi et al., 2009; Staiano et al., 2012). The participants in the post-intervention assessment in the studies faced with a situation that they had experienced previously (pre-intervention assessment). These types of evaluations are not suitable for the clinical trials on EF, because EF assessment tools should examine the cognitive components in new situations. After the first use, neuropsychological tools are not new to the experimental subjects (Jurado & Rosselli, 2007). In addition, these assessment tools are highly structured within the examination; thus, it is impossible to assess skills such as goal setting and decision-making. BRIEF reports EF skills from parents in a natural and unstructured environment of a child’s life,; therefore, it is possible to investigate all aspects of EF (Jurado & Rosselli, 2007).

The BRIEF in the current study measured the views of parents on the student’s executive performance in various everyday activities (Gioia et al., 2000). The significant changes in EF found by this method indicated that the changes in EF skills were generalized to different life situations. It is worth mentioning the following facts from the field of neuroscience: 1) Long duration, familiar, and repetitive tasks can lead to stable networks and synaptic changes in the brain and increase the sensitivity of neurons to the stimuli; 2) The meaningful nature and repetition of tasks enhances nonlinear dynamic changes in brain processing; and 3) When a skill is learned in this way, encoding and retrieving it from the implicit memory would be very easy (Li & Gilbert, 2009).

In the light of these statements, it is likely that the play-based therapy method employed in the present study had the characteristics of familiarity, meaningfulness, and repetition of activities, which can lead to enhanced nonlinear dynamic processing in the brain; in this case, the findings of BRIEF as a parent-report instrument demonstrated that the information recorded in the nervous system could be easily used by the subjects in different real life situations.

An important implication of the present study was that the playful approach in the treatment of dysexecutive function in students with learning disabilities helped clinicians to access the outcomes generalizable to real life. The present study provided preliminary evidence to consider EF as an executive occupational performance instead of cognitive components in assessment and intervention.

A limitation of the current study was that participants were not blind to the group allocation; the intervention was long-term and it was necessary to explain the whole process of study at the recruitment stage to allow the-parents to make an informed decision on their child’s participation in the study. In addition, the initial randomization was not implemented completely, because 7 subjects refused to participate in the study after randomization (Figure 1); however, using MANCOVA in the data analysis, initial differences between the 2 groups were accounted for, and any further differences between the groups were most likely the outcome of the play-based intervention. Furthermore, a follow-up study helps to determine the long-term effects of such interventions.

As Shaheen (2014) reported that play-based interventions have longer lasting effects, it is expected that the follow-up study demonstrates the long-term effectiveness of the treatment. In terms of future research, it is recommend to compare the effects of play- and non-play-based interventions on EF. Due to the relationships between EF and educational skills, it is recommended to investigate the effects of the intervention on the educational skills of students with SLD such as reading, writing, and mathematics. Even if these students had comorbid disorders, dysexecutive function was the result of SLD rather than comorbid disorders. This showed the strong connection between the processing and executive centers of the brain. It demonstrated that the intervention described in the current study could potentially improve the academic skills in students with SLD.

In summary, it can be said that play-based therapy can increase metacognitive and behavioral regulation skills of EF in students with SLD. These findings showed that the effect of a play-based approach on EF skills was higher than that of the educational approaches. Significant changes measured through a parent-report tool about EF skills in a real-life environment showed that the abilities obtained in the therapy sessions were generalized to natural environments. The current study can help professionals in the cognitive training of students with SLD.
